# Associations between Ethylene Oxide Exposure and Liver Function in the US Adult Population

**DOI:** 10.3390/toxics12080551

**Published:** 2024-07-30

**Authors:** Shanshan Li, Jinzhou Wang, Dengliang Lei, Dadi Peng, Kezhen Zong, Kaili Li, Zhongjun Wu, Yanyao Liu, Zuotian Huang

**Affiliations:** 1 First Affiliated Hospital of Chongqing Medical University, Chongqing 400000, China; 2022110196@stu.cqmu.edu.cn (S.L.); ldldoctor@126.com (D.L.); dadi_peng@163.com (D.P.); zongkezhen23@163.com (K.Z.); 2019110257@stu.cqmu.edu.cn (K.L.); wzjtcy@126.com (Z.W.); 2 Affiliated Hospital of North Sichuan Medical College, Nanchong 637000, China; wjz19990819@163.com

**Keywords:** ethylene oxide, NHANES, liver function test

## Abstract

Background: Ethylene oxide, a reactive epoxy compound, has been widely used in various industries for many years. However, evidence of the combined toxic effects of ethylene oxide exposure on the liver is still lacking. Methods: We analyzed the merged data from the National Health and Nutrition Examination Survey (NHANES) from 2013 to 2016. Ultimately, 4141 adults aged 18 and over were selected as the sample. We used linear regression to explore the association between blood ethylene oxide and LFT indicators. Results: The weighted linear regression model showed that HbEO is positively correlated with ALP (*β* = 2.61, 95% CI 1.97, 3.24, *p* < 0.0001), GGT (*β* = 5.75, 95% CI 4.46, 7/05, *p* < 0.0001), ALT (*β* = 0.50, 95% CI 0.09, 0.90, *p* = 0.0158), and AST (*β* = 0.71, 95% CI 0.44, 0.98, *p* < 0.0001) and negatively correlated with TBIL (*β* = −0.30, 95% CI −0.43, −0.16, *p* < 0.0001). Conclusions: Ethylene oxide exposure is significantly associated with changes in liver function indicators among adults in the United States. Future work should further examine these relationships.

## 1. Introduction

As the largest synthetic organ in the human body, the liver participates in physiological functions such as plasma protein synthesis, gluconeogenesis and glycogen storage, cholesterol metabolism, bile acid synthesis, and detoxification [1,2]. In recent decades, liver diseases, such as non-alcoholic fatty liver disease, alcoholic liver disease, and viral hepatitis, have become one of the leading causes of death and illness worldwide, with approximately 2 million people dying from these diseases each year [3,4]. Since the last century, liver function tests (LFTs) have been the primary means of diagnosing liver disease [5]. During the progression of liver diseases, when hepatocyte necrosis or liver cell membrane damage occurs, the serum levels of alanine aminotransferase (ALT), aspartate aminotransferase (AST), and total bilirubin (TBIL) often increase [6,7]. Alkaline phosphotase (ALP) and gamma-glutamyl transferase (GGT) are commonly used to identify cholestasis in medical research [8,9]. Increasing evidence suggests that many pollutants and chemical contaminants in the environment may cause liver damage and increase the risk of liver disease [10,11,12].

Ethylene oxide (EO), a reactive epoxy compound, is widely utilized in the production of plastics and surfactants and in the sterilization of fragrances, cosmetics, and medical devices. The primary marker for EO exposure is the ethylene oxide adduct in hemoglobin (HbEO), measured using a modified Edman reaction. EO exposure predominantly occurs through exogenous pathways, including inhalation and ingestion, impacting occupational, environmental, and consumer health. Additionally, EO is endogenously produced in the body via cytochrome P450 2E1-mediated oxidation of ethylene [13,14,15]. However, despite its widespread use, EO has associated health risks that have garnered significant attention. Existing animal studies have revealed that high-dose EO exposure leads to damage across multiple systems, including the respiratory, hematological, nervous, reproductive, and renal systems [16,17,18,19]. More alarmingly, the International Agency for Research on Cancer has classified the alkylating agent EO as a Group 1 human carcinogen [20]. In recent years, beyond the aforementioned health risks, large-scale cross-sectional studies have also suggested potential links between EO and chronic kidney disease, asthma, dyslipidemia, and hypertension [21,22,23,24]. However, the relationship between EO exposure and liver damage remains unclear.

We conducted a cross-sectional study using data from the NHANES 2013–2016 to investigate the relationship between ethylene oxide exposure and liver function damage among adults in the United States. Initially, 20,146 participants were enrolled. A total of 4141 participants were included in our final analysis after excluding individuals with missing data on liver function tests (n = 7338) and those with missing data on ethylene oxide (n = 8667).

## 2. Materials and Methods

### 2.1. Study Population

**Figure 1:**
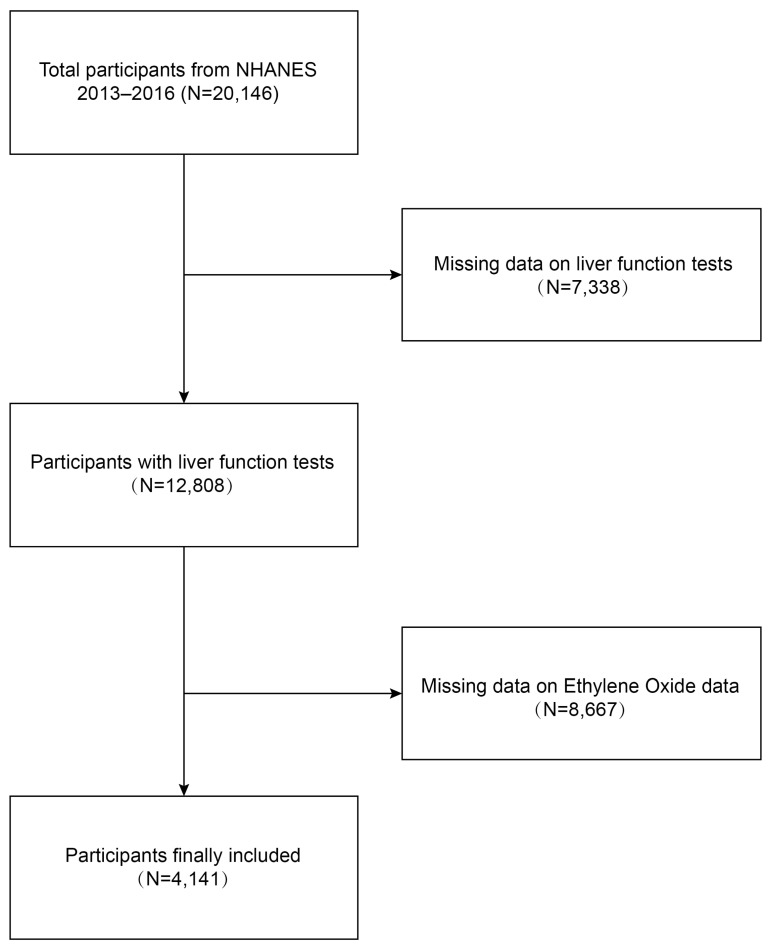
Process map for sample collection from NHANES.

Data were obtained from NHANES, a national population-based cross-sectional survey to collect information about the potential health risk factors and nutrition status of non-institutionalized US civilians, which was conducted by the National Center for Health Statistics (NCHS) [25]. A complex, stratified, multistage probability cluster sampling design was employed to recruit a representative sample of the whole US population. The specific research method is as follows: Counties are stratified into 15 distinct clusters based on shared characteristics for the purposes of the NHANES. From each cluster, one representative county is selected annually to participate in the survey. Subsequently, within each selected county, 20 to 24 neighborhoods are identified as smaller sampling units. Comprehensive lists of all residential units within these neighborhoods are compiled, from which approximately 30 households per neighborhood are randomly chosen for inclusion in the study. NHANES personnel initiate contact with these selected households to administer a preliminary questionnaire, which gathers essential demographic data including the age, race, and gender of all household members. A computerized random selection algorithm is then employed to determine the final inclusion of individuals from these households into the survey cohort, with the selection process potentially including some, all, or none of the household members. All NHANES participants provided informed signed agreement, and approval was obtained from the Research Ethics Committee of the National Centre for Health Statistics. The detailed NHANES study design and data are publicly available at https://www.cdc.gov/nchs/nhanes/ (accessed on 25 June 2024). Participants received a standardized in-home interview and health examination at mobile examination centers to assess their medical and physiological status, and laboratory tests were conducted to collect their laboratory data. Our study was based on two NHANES survey cycles from 2013 to 2014 and from 2015 to 2016, knowing these two cycles include data on both HbEO and liver function tests. The inclusion criteria for the participants were as follows: (1) individuals over 18 years old, (2) individuals with comprehensive demographic information, and (3) individuals with complete ethylene oxide and liver function tests. The exclusion criteria were as follows: (1) individuals lacking ethylene oxide laboratory data, (2) individuals without liver function test data, (3) individuals younger than 18 years old. A total of 20,146 participants were enrolled at first. Individuals missing data on liver function tests (n = 7338) and ethylene oxide (n = 8667) were then excluded, and 4141 participants were included in our final analysis (Figure 1).

### 2.2. Assessment of Hemoglobin Ethylene Oxide Levels

The measurement of HbEO in this investigation was conducted according to the NHANES Laboratory/Medical Technologist Procedures Manual, which may be found at https://wwwn.cdc.gov/Nchs/Nhanes/2013-2014/ETHOX_H.htm (accessed on 25 June 2024). The collection, processing, and transportation of specimens were conducted in accordance with established protocols.

The Division of Laboratory Sciences of the National Center for Environmental Health (NCEH) has been assigned the responsibility of measuring HbEO. Detailed information about the laboratory analysis techniques can be found on the corresponding webpage. The researchers utilized the modified Edman reaction and high-performance liquid chromatography coupled with tandem mass spectrometry to identify the presence of ethylene oxide (EO) adducts in hemoglobin. Specifically, they focused on detecting two types of adducts: N-(2-carbamoyl-ethyl) valine EO adducts (CEVs) and N-(2-hydroxycarbamoyl-ethyl) valine EO adducts (HEVs). The levels of HbEO were quantified and expressed in picomoles per gram of hemoglobin (pmol/g Hb). The assays’ accuracy and precision were in line with the quality control and quality assurance standards set by the NCEH Laboratory Science Division (CDC 2023).

### 2.3. Measurement of Liver Function

Serum LFTs were measured using various methods utilizing a Beckman Coulter UniCel DxC800 Synchron Clinical System (Beckman Coulter, Inc., Brea, CA, USA). The activities of both ALT and ALP were determined using the kinetic rate method commonly employed in medical research. The enzymatic rate method was used to determine the activities of AST and GGT. The TBIL serum level was measured using a timed-endpoint Diazo method.

### 2.4. Covariates

Potential covariates that might confound the association between HbEO and LFTs were summarized in the multivariable-adjusted models. Covariates in our study included gender (male/female), age (<50 and ≥50 years), race, education level, poverty-to-income ratio (PIR), total cholesterol level (mg/dL), HDL level (mmol/L), body mass index (BMI), smoking status (smoking or not), diabetes, aspirin, alcohol, acetaminophen, statins, hepatitis B, and hepatitis C.

PIR was estimated as the ratio of family income to the poverty threshold, and participants were divided into low-income (PIR < 1.3), middle-income (PIR = 1.30–3.50), and high-income (PIR ≥ 3.50) groups. BMI was categorized as <25, 25–29.9, and ≥30 kg/m^2^, which correspond to normal weight, overweight, and obese population for all participants. All detailed measurement processes for study variables are publicly available at www.cdc.gov/nchs/nhanes/ (accessed on 25 June 2024).

### 2.5. Statistical Analysis

All statistical analyses were conducted according to Centers for Disease Control and Prevention (CDC) guidelines, and an appropriate NHANES sampling weight was applied, accounting for the complex multistage cluster survey design in the analysis. Continuous variables are presented as means with standard deviation, and categorical variables are presented as a percentage. Either a weighted Student’s *t*-test (for continuous variables) or weighted chi-square test (for categorical variables) was used to evaluate the differences in groups divided by HbEO (quartiles). Multivariate logistic regression models were employed to explore the independent relationship between HbEO and liver function tests in three different models. In model 1, no covariates were adjusted. Model 2 was adjusted for gender, age, and race. Model 3 was adjusted for gender, age, race, education level, poverty-to-income ratio, HDL, total cholesterol, BMI, smoking status, diabetes, aspirin, alcohol, statins, hepatitis B, and hepatitis C. Smooth curve fitting (penalized spline method) and weighted generalized additive model (GAM) regression were conducted to further assess the nonlinear relationship between HbEO and liver function tests. Subgroup analysis stratified by gender, age, BMI, diabetes, and education level was also performed via stratified multivariate regression analysis. In addition, an interaction term was added to test the heterogeneity of associations between the subgroups using the log likelihood ratio test model. *p* < 0.05 was considered statistically significant. All analyses were performed using Empower version 4.1 (www.empowerstats.com; X&Y solutions, Inc., Boston, MA, USA) and R version 3.4.3 (http://www.R-project.org, The R Foundation, Vienna, Austria).

## 3. Results

### 3.1. Baseline Characteristics of Participants

Weighted demographic baseline characteristics of included participants are shown in Table 1. A total of 4141 participants were included in our study, of whom 50.02% were male and 49.98% were female, with the average age of 43.68 ± 18.99 years. The mean of log2 HbEO was 4.74 ± 1.47, and the ranges of log2 HbEO for quartiles 1–4 were 2.54–3.91, 3.91–4.37, 4.37–5.18, and 5.18–10.35, respectively. In total, compared to those in quartile 1, participants in quartile 4 had lower rates of ALP and TBIL and a higher rate of GGT. In addition, we found statistically significant differences by age, gender, race, education level, PIR, BMI, diabetes, smoking, serum HDL, serum cholesterol, alcohol, acetaminophen, statins, hepatitis B, and hepatitis C (all *p* < 0.05) among log2 HbEO quartiles. Similarly, the results in the population baseline table remained consistent across quartiles of HbEO that had not undergone logarithmic transformation (Supplement Table S1).

### 3.2. Multiple Linear Regression Associations of Ethylene Oxide with LFTs in Adults

**Figure 2:**
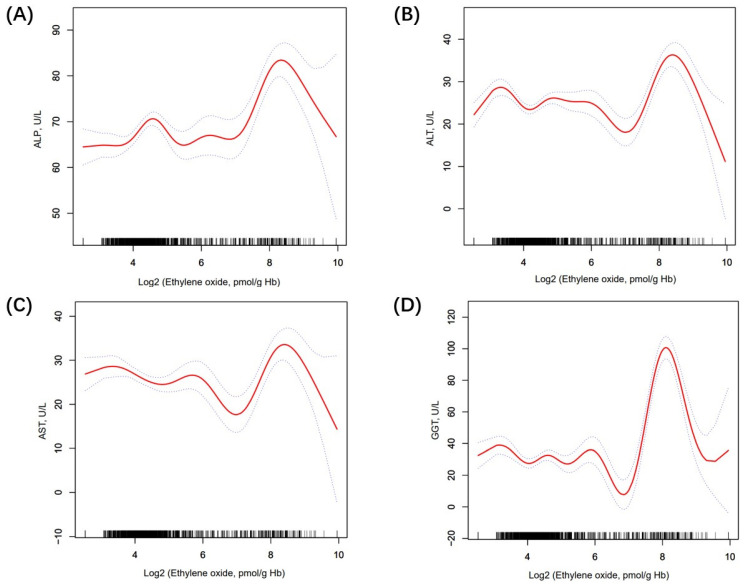
The nonlinear relationship between LFTs and log2-HbEO. (**A**) The association between ALP and HbEO; (**B**) The association between ALT and HbEO; (**C**) The association between AST and HbEO; (**D**) The association between GGT and HbEO. The solid line and dashed lines represent the fitted line and 95% confidence interval, respectively.

Table 2 shows the association between ethylene oxide and LFTs. Firstly, the log2-transformed HbEO was utilized as a continuous variable to investigate its correlation with liver function. In model 1, we found that ALT (*β* 0.69, 95% CI 0.40, 0.99, *p* < 0.0001), AST (*β* 0.71, 95% CI 0.44, 0.98, *p* < 0.0001), and GGT (*β* 1.63, 95% CI 0.80, 2.45, *p* < 0.001) were positively related to HbEO. In contrast, TBIL (*β* −0.42, 95% CI −0.50, −0.34, *p* < 0.0001) was negatively linked with HbEO. In model 2, we found that ALP (*β* = 2.61, 95% CI 1.97, 3.24, *p* < 0.0001), GGT (*β* = 5.75, 95% CI 4.46, 7/05, *p* < 0.0001), and ALT (*β* = 0.50, 95% CI 0.09, 0.90, *p* = 0.0158) had a positive relationship with HbEO. However, TBIL (*β* = −0.30, 95% CI −0.43, −0.16, *p* < 0.0001) was negatively linked with HbEO. Then, sensitivity analysis was conducted, treating log2-transformed HbEO as a categorical variable (quartiles). In the fully adjusted model (model 2), compared with the lowest HbEO quartile (quartile 1), participants in the top HbEO quartile had 8.48 IU/L higher ALP, 15.06 IU/L higher GGT, 2.60 IU/L lower AST, and 1.19 IU/L lower TBIL than those in the HbEO quartile, and the *P* values for trends were <0.0001, <0.0001, 0.0695, <0.0001, correspondingly. However, the association between ALT (*β* −2.41, 95% CI −7.83, 3.01, *p* = 0.3853) and HbEO quartiles met statistical significance only in model 1. Moreover, smooth curve fitting exhibited a non-linear relationship between LFTs and HbEO (Figure 2). We further calculated the inflection points for ALP, ALT, AST, and GGT to be 6.73, 6.58, 6.62, and 6.08, respectively. To the left of the inflection point, a positive relationship between ALT, AST, GGT, and HbEO (*β* = −0.41, 95% CI: −1.16, 0.35, *p* = 0.2884; *β* = −1.82, 95% CI: −2.67, −0.97, *p* < 0.0001; *β* = −2.10, 95% CI: −4.42, 0.23, *p* = 0.0769) was detected. Conversely, to the right of the inflection point, a negative relationship between ALT, AST, GGT, and HbEO (*β* = 3.43, 95% CI: 1.83, 5.03, *p* < 0.0001; *β* = 4.02, 95% CI: 2.16, 5.89, *p* < 0.0001; *β* = 17.81, 95% CI: 14.38, 21.23, *p* < 0.0001) was observed. After the adjustment of covariates, the logarithmic likelihood ratio test *P* value was <0.001 (Supplement Table S2). However, in the quartiles of HbEO without Log2 transformation, the linear regression results did not exhibit statistically significant differences (Supplement Table S3).

### 3.3. Subgroup Analysis

In order to evaluate whether the association between ethylene oxide and LFTs was consistent in the overall population and find the potential different population settings, we conducted subgroup analysis and an interaction test stratified by gender, age, education level, BMI, diabetes, and smoking (Table 3). In the male group, there was a negative correlation between ALT and GGT (*β* = −0.75, 95% CI −1.35, −0.14, *p* < 0.0001; *β* = −0.70, 95% CI −2.59, 1.19, *p* < 0.0001) and a robust positive correlation between ALP and HbEO concentrations (*β* = 3.81, 95%CI 2.86, 4.76, *p* < 0.0001). In the younger subgroup stratified by age, ALP and TBIL exhibited positive correlation with HbEO (*β* = 5.56, 95% CI 3.78, 7.33, *p* = 0.0002; *β* = 0.05, 95% CI −0.21, 0.30, *p* = 0.0036), while ALT and GGT showed negative association (all *p* < 0.05). Regarding subgroup analyses stratified by education level, ALT, GGT, and HbEO in the high school groups showed a negative connection (*β* = −1.10, 95% CI −2.05, −0.16, *p* = 0.0245; *β* = −0.41, 95% CI 3.35, 2.52, *p* = 0.0007). With regard to subgroup analyses stratified by BMI, GGT was strongly positive linked with HbEO in the high BMI group (*β* = 2.32, 95% CI 1.20, 3.44, *p* = 0.0227), while AST and GGT showed negative association (*β* = −0.88, 95% CI −1.42, −0.34, *p* = 0.0016). With reference to diabetes-stratified subgroup analyses, there was a positive correlation between ALT and HbEO in the diabetes groups (*β* = 1.49, 95% CI 0.81, 2.18, *p* < 0.0001). Furthermore, GGT showed positive correlation with HbEO in smokers compared with non-smokers (*β* = 5.51, 95% CI 4.09, 6.94, *p* < 0.0001). Taken together, the substantial connection with the p for interaction suggested that this association between liver function and HbEO showed dependence on gender, age, education level, BMI, diabetes, and smoking (*p* for interaction < 0.05).

## 4. Discussion

In this nationally representative study, we primarily examined the impact of ethylene oxide (HbEO) on liver function tests (LFTs) in adults, revealing significant associations between HbEO levels and GGT, TBIL, and ALP. Specifically, elevated EtO exposure was correlated with increasing ALP, ALT, and GGT, while, intriguingly, negative correlation emerged with TBIL. Additionally, the non-linear relationship between HbEO and ALP, ALT, AST, and GGT changes direction significantly beyond the identified inflection points of 6.73, 6.58, 6.62, and 6.08 pmol/g Hb. These findings provide new insights into the potential impact of ethylene oxide on liver function, underscoring the necessity of further research into the links between environmental exposure and liver health.

To our knowledge, this is the first study to examine the association between ethylene oxide and liver function indexes in US adults. Ethylene oxide, a reactive epoxy compound, is commonly used for sterilizing materials such as food, spices, and medical devices [26]. Previous studies have found that the health impacts of ethylene oxide are primarily due to its carcinogenicity, mutagenicity, and toxic effects [27,28,29]. Although the health impacts of ethylene oxide mainly affect workers in medical device sterilization, the general population’s risk of exposure increases through activities such as renovations, smoking, and living near facilities that use ethylene oxide [30,31]. Several cross-sectional surveys based in the United States have discovered a significant correlation between exposure to ethylene oxide and conditions such as kidney stones, depression, asthma, and chronic kidney disease in the general population [22,32,33]. However, no reports on its impact on the liver have been published. In this study, we utilized multiple LFTs to quantitatively assess the liver damage in participants. The results indicate a positive correlation between exposure to ethylene oxide and liver function markers GGT and ALP and a negative correlation with TBIL and ALT, consistent with recent studies on the impact of environmental toxins on liver function [34,35]. The nonlinear relationship between liver function tests (LFTs) and hemoglobin-bound ethylene oxide (HbEO) exhibits a threshold effect: when the HbEO concentration is below the inflection point, ALP, ALT, AST, and GGT levels increase with the rise in HbEO levels. However, when HbEO concentration exceeds the inflection point, ALP, ALT, AST, and GGT levels begin to decrease. This observed threshold effect suggests that at lower HbEO levels, the liver mitigates oxidative stress by augmenting liver metabolite production, thereby promoting glutathione turnover to manage the elevated oxidative load. Nonetheless, when HbEO concentrations surpass the liver’s oxidative stress management capacity, the ability of HbEO to produce liver metabolites and regulate oxidative stress becomes overwhelmed, leading to a decline in ALP, ALT, AST, and GGT levels. This pattern underscores the liver’s adaptive response to oxidative stress, with glutathione acting as a biomarker for liver metabolism and oxidative stress levels. Subgroup analysis revealed that the impact of ethylene oxide exposure on liver function remains consistent across various subgroups. Further, subgroup analysis underscored the interaction between HbEO exposure and conditions such as diabetes and obesity, observing different impacts on liver enzyme levels. These interactions highlight the complex interplay between metabolic health and exposure to environmental toxins, suggesting that individuals with metabolic disorders might be more sensitive to the hepatotoxic effects of HbEO [36]. The variation in TBIL levels between smokers and non-smokers exposed to HbEO also suggests a potential modulatory effect of smoking on liver function and its response to environmental toxins [37].

The potential mechanisms linking ethylene oxide exposure to liver damage remain unclear and warrant further investigation. Our study findings indicate a positive correlation between ethylene oxide exposure and certain liver injuries, suggesting that increased exposure could potentially adversely affect liver health. Several possible mechanisms and genes could underpin these associations. Firstly, EO generates reactive oxygen species (ROS), resulting in oxidative stress and DNA damage. This oxidative stress disrupts cellular functions and leads to hepatotoxicity [38]. Additionally, EO can cause epigenetic modifications such as DNA methylation and histone modification, which alter gene expression patterns and contribute to liver dysfunction [39]. EO exposure induces an inflammatory response in the liver, characterized by immune cell activation and pro-inflammatory cytokine release, which exacerbates liver damage [40]. Moreover, EO disrupts the hepatic metabolic pathways crucial for detoxification, leading to the accumulation of toxic intermediates and further liver damage [41]. The exposure also activates apoptotic and necrotic pathways in liver cells, causing significant tissue damage and functional decline [38]. The key genes affected by EO include TP53, vital for DNA repair and apoptosis regulation, and cytochrome P450 enzymes such as CYP2E1 and CYP3A4, which are involved in EO metabolism [42,43]. EO impacts the Nrf2 signaling pathway, reducing antioxidant capacity and increasing oxidative stress. Additionally, genes encoding pro-inflammatory cytokines IL-6 and TNF-α and apoptosis regulators BAX and BCL-2 are modulated by EO, promoting chronic inflammation and apoptotic cell death [44]. Molecular biomarkers associated with EO exposure and liver function include 8-hydroxy-2′-deoxyguanosine (8-OHdG), indicating oxidative DNA damage, and reduced glutathione (GSH) levels, reflecting oxidative stress. Elevated liver enzymes ALT and AST signify liver damage, while increased serum levels of IL-6 and TNF-α indicate inflammation. Dysregulated microRNAs, such as miR-122 and miR-192, serve as biomarkers for EO-induced liver toxicity [45,46].

Our study boasts several advantages. Firstly, by utilizing nationwide representative data and a robust statistical framework, we provide insights into the exposure to ethylene oxide and its association with liver function, especially by identifying the inflection point in the ALP/ALT/AST/GGT–HbEO relationship. This offers a new dose–response understanding of the hepatotoxic effects of EtO. However, certain limitations must be considered. The cross-sectional design limits the ability to infer causality between HbEO exposure and changes in liver function tests. Additionally, relying on self-reported data for certain demographic and lifestyle factors could introduce bias. Future research should focus on longitudinal designs to better elucidate the temporal dynamics between HbEO exposure and liver function, as well as mechanistic studies to understand the underlying biological pathways.

## 5. Conclusions

In summary, our study demonstrates a significant association between high levels of HbEO exposure and liver function tests (LFTs). Moreover, our data suggest that exposure to ethylene oxide may be linked to liver damage. Given the potential limitations of the current study, further longitudinal research is needed to validate the hepatotoxicity of ethylene oxide exposure and explore potential mechanisms.

## Figures and Tables

**Table 1:** Baseline characteristics of the study population.

Characteristic	Q1	Q2	Q3	Q4	*p*-Value
	N = 1029	N = 1035	N = 1041	N = 1036	
Age (years)	18.98	19.75	20.71	16.32	<0.0001
<50	44.54	42.19	38.76	51.47	
≥50	55.46	57.81	61.24	48.53	
Gender					<0.0001
Male	42.87	45.57	56.95	49.52	
Female	57.13	54.43	43.05	50.48	
Race, n (%)					<0.0001
Mexican American	7.29	8.95	8.88	5.70	
Other Hispanic	5.01	4.69	5.09	4.02	
Non-Hispanic White	76.60	69.60	62.78	69.20	
Non-Hispanic Black	6.43	9.78	11.76	14.43	
Other Race	4.68	6.98	11.49	6.66	
Education Level, n (%)					<0.0001
Less than high school	10.62	13.01	12.31	22.16	
High school	19.41	19.07	31.87	28.20	
More than high school	69.97	67.92	55.82	49.64	
PIR					<0.0001
<1.3	14.94	18.22	27.42	41.25	
1.3–3.5	40.32	38.11	35.15	36.26	
≥3.5	44.74	43.66	37.42	22.49	
BMI (kg/m^2^)					<0.0001
<25	26.52	21.58	28.92	31.37	
>25	73.48	78.42	71.08	68.63	
Smoking, n (%)					<0.0001
Yes	33.75	31.00	45.25	88.25	
No	66.25	69.00	54.75	11.75	
Diabetes, n (%)					<0.0001
Yes	12.36	18.95	27.76	15.29	
No	87.64	81.05	72.24	84.71	
Alcohol, n (%)					<0.0001
Yes	78.24	66.57	78.18	83.37	
No	21.76	33.43	21.82	16.63	
Aspirin, n (%)					0.1284
Yes	68.58	73.78	74.05	59.80	
No	31.42	26.22	25.95	40.20	
Acetaminophen					0.0021
Yes	0.61	1.22	0.58	1.71	
No	99.39	98.78	99.42	98.29	
Statins, n (%)					0.0121
Yes	82.65	87.93	82.35	83.76	
No	17.35	12.07	17.65	16.24	
Hepatitis B, n (%)					<0.0001
Yes	1.25	1.80	1.64	3.55	
No	98.75	98.20	98.36	96.45	
Hepatitis C, n (%)					<0.0001
Yes	1.06	0.24	1.46	6.03	
No	98.94	99.76	98.54	93.97	
AST (U/L)	26.04 ± 10.41	25.38 ± 16.32	24.85 ± 10.15	30.09 ± 25.75	<0.0001
ALT (U/L)	24.82 ± 15.66	24.48 ± 14.54	24.93 ± 14.56	28.62 ± 26.88	<0.0001
GGT (U/L)	26.29 ± 27.69	25.53 ± 32.51	25.23 ± 21.24	46.95 ± 91.90	<0.0001
ALP (U/L)	69.11 ± 38.62	71.46 ± 40.62	72.11 ± 40.67	72.64 ± 30.06	0.0242
TBIL (μmol/L)	10.81 ± 6.88	10.44 ± 4.99	10.53 ± 4.48	9.38 ± 4.14	<0.0001
HDL (mmol/L)	54.77 ± 18.00	52.29 ± 14.52	51.35 ± 15.36	50.23 ± 15.83	<0.0001
Total cholesterol (mmol/L)	186.89 ± 40.65	181.75 ± 43.00	179.66 ± 40.33	187.98 ± 42.82	<0.0001

Continuous variables are presented as means ± standard deviation, and the *p* value was calculated using the weighted linear regression model. Categorical variables are displayed as a percentage.

**Table 2:** The association between log2-transform HbEO and the liver function test.

LFTs	Model	Continuous log2-Transformed EO	Quartile 1 *β* (95% CI)	Quartile 2 *β* (95% CI)	Quartile 3 *β* (95% CI)	Quartile 4 *β* (95% CI)	*p* for Trend
ALP	Crude	1.00 (0.40, 1.60) 0.0012	0.00 (Ref.)	2.35 (−0.06, 4.75) 0.0556	3.00 (0.47, 5.53) 0.0201	3.53 (1.06, 6.00) 0.0051	0.0179
	Model 1	0.47 (−0.12, 1.06) 0.1169	0.00 (Ref.)	1.98 (−0.36, 4.31) 0.0974	2.97 (0.48, 5.46) 0.0194	1.68 (−0.73, 4.10) 0.1722	0.4080
	Model 2	2.61 (1.97, 3.24) <0.0001	0.00 (Ref.)	0.48 (−1.78, 2.74) 0.6760	3.75 (1.30, 6.20) 0.0027	8.48 (5.75, 11.22) <0.0001	<0.0001
ALT	Crude	0.72 (0.42, 1.01) <0.0001	0.00 (Ref.)	−0.34 (−1.51, 0.84) 0.5754	0.11 (−1.12, 1.35) 0.8573	3.80 (2.59, 5.01) <0.0001	<0.0001
	Model 1	0.69 (0.40, 0.99) <0.0001	0.00 (Ref.)	−0.42 (−1.57, 0.73) 0.4747	−0.73 (−1.96, 0.49) 0.2398	3.65 (2.46, 4.83) <0.0001	<0.0001
	Model 2	0.50 (0.09, 0.90) 0.0158	0.00 (Ref.)	−1.65 (−3.07, −0.24) 0.0223	−0.25 (−1.79, 1.29) 0.7517	0.36 (−1.36, 2.07) 0.6846	0.3020
AST	Crude	0.67 (0.40, 0.94) <0.0001	0.00 (Ref.)	−0.67 (−1.73, 0.40) 0.2187	−1.20 (−2.32, −0.08) 0.0358	4.05 (2.95, 5.14) <0.0001	<0.0001
	Model 1	0.71 (0.44, 0.98) <0.0001	0.00 (Ref.)	−0.67 (−1.72, 0.39) 0.2136	−1.71 (−2.84, −0.59) 0.0028	4.16 (3.07, 5.25) <0.0001	<0.0001
	Model 2	−0.39 (−0.89, 0.11) 0.1242	0.00 (Ref.)	−2.54 (−4.29, −0.78) 0.0047	−3.17 (−5.07, −1.26) 0.0011	−2.60 (−4.73, −0.48) 0.0165	0.0695
GGT	Crude	4.53 (3.72, 5.35) <0.0001	0.00 (Ref.)	−0.75 (−3.98, 2.48) 0.6476	−1.06 (−4.46, 2.35) 0.5430	20.66 (17.34,23.98) <0.0001	<0.0001
	Model 1	4.62 (3.80, 5.44) <0.0001	0.00 (Ref.)	−1.09 (−4.31, 2.12) 0.5049	−2.70 (−6.13, 0.72) 0.1222	20.81 (17.49,24.14) <0.0001	<0.0001
	Model 2	5.75 (4.46, 7.05) <0.0001	0.00 (Ref.)	−3.84 (−8.43, 0.76) 0.1018	−1.33 (−6.32, 3.66) 0.6016	15.06 (9.50, 20.63) <0.0001	<0.0001
TBIL	Crude	−0.39 (−0.48, −0.31) <0.0001	0.00 (Ref.)	−0.38 (−0.71, −0.04) 0.0302	−0.29 (−0.64, 0.07) 0.1150	−1.43 (−1.78, −1.08) <0.0001	<0.0001
	Model 1	−0.42 (−0.50, −0.34) <0.0001	0.00 (Ref.)	−0.42 (−0.75, −0.09) 0.0125	−0.62 (−0.98, −0.27) 0.0005	−1.55 (−1.89, −1.21) <0.0001	<0.0001
	Model 2	−0.30 (−0.43, −0.16) <0.0001	0.00 (Ref.)	0.18 (−0.29, 0.65) 0.4593	0.10 (−0.41, 0.61) 0.7009	−1.19 (−1.76, −0.62) <0.0001	<0.0001

Crude model: no covariates were adjusted. Model 1: age, gender, and race were adjusted. Model 2: age, gender, race, educational level, BMI, family income-to-poverty ratio, moderate activities, diabetes status, aspirin, acetaminophen, alcohol, statins, hepatitis B, hepatitis C, smoking, HDL level, and total cholesterol were adjusted.

**Table 3:** Subgroup analysis of the association between log2-HbEO and LFTs.

Subgroup	ALP [*β* (95% CI)]	*p* for Interaction	ALT [*β* (95% CI)]	*p* for Interaction	AST [*β* (95% CI)]	*p* for Interaction	GGT [*β* (95% CI)]	*p* for Interaction	TBIL [*β* (95% CI)]	*p* for Interaction
Gender		<0.0001		<0.0001		0.2503		<0.0001		0.7400
Male	3.81 (2.86, 4.76)		−0.75 (−1.35, −0.14)		−0.36 (−1.12, 0.40)		−0.70 (−2.59, 1.19)		−0.22 (−0.43, −0.02)	
Female	0.87 (0.00, 1.74)		1.23 (0.68, 1.78)		−0.96 (−1.65, −0.27)		8.83 (7.11, 10.56)		−0.27 (−0.45, −0.09)	
Age		0.0002		0.0086		0.5331		0.0002		0.0036
<50	5.56 (3.78, 7.33)		−0.86 (−1.98, 0.27)		−0.91 (−2.30, 0.49)		−0.47 (−4.11, 3.17)		0.05 (−0.21, 0.30)	
≥50	1.97 (1.26, 2.67)		0.75 (0.30, 1.19)		−0.44 (−0.99, 0.12)		7.04 (5.59, 8.48)		−0.38 (−0.53, −0.23)	
Education level		0.0403		0.0245		0.5037		0.0007		<0.0001
Less than high school	1.95 (0.56, 3.34)		0.03 (−0.87, 0.92)		−1.68 (−2.80, −0.56)		4.70 (1.92, 7.48)		−0.97 (−1.26, −0.67)	
High school	3.47 (2.01, 4.94)		−1.10 (−2.05, −0.16)		−1.18 (−2.36, −0.00)		−0.41 (−3.35, 2.52)		0.14 (−0.18, 0.45)	
More than high school	1.33 (0.49, 2.16)		0.39 (−0.15, 0.93)		−0.92 (−1.59, −0.24)		6.04 (4.37, 7.71)		−0.18 (−0.36, 0.00)	
BMI		0.2370		0.2795		0.0016		0.0039		0.9680
<25	1.14 (−0.69, 2.97)		−0.06 (−1.21, 1.09)		1.56 (0.13, 3.00)		0.44 (−3.26, 4.14)		−0.29 (−0.67, 0.10)	
≥25	2.31 (1.62, 2.99)		0.61 (0.18, 1.04)		−0.88 (−1.42, −0.34)		6.22 (4.83, 7.61)		−0.29 (−0.44, −0.15)	
Diabetes status		<0.0001		<0.0001		0.0253		0.0597		0.7444
Yes	0.21 (−0.85, 1.28)		1.49 (0.81, 2.18)		−1.35 (−2.20, −0.51)		6.85 (4.65, 9.05)		−0.37 (−0.60, −0.14)	
No	3.53 (2.74, 4.32)		−0.37 (−0.88, 0.13)		−0.16 (−0.79, 0.47)		4.25 (2.62, 5.88)		−0.32 (−0.49, −0.15)	
Smoking		0.3743		0.2015		0.9654		<0.0001		0.0048
Yes	1.89 (1.17, 2.60)		0.40 (−0.06, 0.86)		−0.68 (−1.25, −0.11)		5.51 (4.09, 6.94)		−0.46 (−0.61, −0.31)	
No	2.83 (0.86, 4.79)		−0.46 (−1.71, 0.79)		−0.72 (−2.29, 0.85)		−4.05 (−7.96, −0.15)		0.15 (−0.25, 0.55)	

Adjusted for age, gender, race, educational level, BMI, family income-to-poverty ratio, moderate activities, diabetes status, aspirin, acetaminophen, alcohol, statins, hepatitis B, hepatitis C, smoking, HDL level, and total cholesterol.

## Data Availability

The original data presented in this study are openly available in the National Health and Nutrition Examination Survey at https://www.cdc.gov/nchs/nhanes/index.htm (accessed on 25 June 2024).

## Supplementary Materials

The following supporting information can be downloaded at: https://www.mdpi.com/article/10.3390/toxics12080551/s1, Table S1: Baseline characteristics of the study population, Table S2: The threshold effect of log2-HbEO on LFTs using a two-stage phased regression model, Table S3: The association between HbEO and the liver function test.
