# Supplementary material for: Associations between Ethylene Oxide Exposure and Liver Function in the US Adult Population
Source: Toxics. 2024 Jul 30;12(8):551. doi: 10.3390/toxics12080551 (PMC11358929; doi:10.3390/toxics12080551)
Supplement: Supplementary file 1 [file toxics-12-00551-s001.zip › Table S1.pdf]

**Table S1** Baseline characteristics of the study population.

| Characteristic | Q1<br>N=1029 | Q2<br>N=1035 | Q3<br>N=1041 | Q4<br>N=1036 | *P*-value |
|---|---|---|---|---|---|
| Age(years) | 18.98 | 19.75 | 20.71 | 16.32 | <0.0001 |
| <50 | 44.54 | 42.19 | 38.76 | 51.47 | |
| ≥50 | 55.46 | 57.81 | 61.24 | 48.53 | |
| Gender | | | | | <0.0001 |
| Male | 42.87 | 45.57 | 56.95 | 49.52 | |
| Female | 57.13 | 54.43 | 43.05 | 50.48 | |
| Race, n(%) | | | | | <0.0001 |
| Mexican American | 7.29 | 8.95 | 8.88 | 5.70 | |
| Other Hispanic | 5.01 | 4.69 | 5.09 | 4.02 | |
| Non-Hispanic White | 76.60 | 69.60 | 62.78 | 69.20 | |
| Non-Hispanic Black | 6.43 | 9.78 | 11.76 | 14.43 | |
| Other Race | 4.68 | 6.98 | 11.49 | 6.66 | |
| Education Level, n(%) | | | | | <0.0001 |
| Less than high school | 10.62 | 13.01 | 12.31 | 22.16 | |
| High school | 19.41 | 19.07 | 31.87 | 28.20 | |
| More than high school | 69.97 | 67.92 | 55.82 | 49.64 | |
| PIR | | | | | <0.0001 |
| <1.3 | 14.94 | 18.22 | 27.42 | 41.25 | |
| 1.3-3.5 | 40.32 | 38.11 | 35.15 | 36.26 | |
| ≥3.5 | 44.74 | 43.66 | 37.42 | 22.49 | |
| BMI（kg/m2） | | | | | <0.0001 |
| <25 | 26.52 | 21.58 | 28.92 | 31.37 | |
| ≥25 | 73.48 | 78.42 | 71.08 | 68.63 | |
| Smoking, n(%) | | | | | <0.0001 |
| Yes | 33.75 | 31.00 | 45.25 | 88.25 | |
| No | 66.25 | 69.00 | 54.75 | 11.75 | |
| Diabetes, n(%) | | | | | <0.0001 |
| Yes | 12.36 | 18.95 | 27.76 | 15.29 | |
| No | 87.64 | 81.05 | 72.24 | 84.71 | |
| Alcohol, n(%) | | | | | <0.0001 |
| Yes | 78.24 | 66.57 | 78.18 | 83.37 | |
| No | 21.76 | 33.43 | 21.82 | 16.63 | |
| Aspirin, n(%) | | | | | 0.1284 |
| Yes | 68.58 | 73.78 | 74.05 | 59.80 | |
| No | 31.42 | 26.22 | 25.95 | 40.20 | |
| Acetaminophen | | | | | 0.0021 |
| Yes | 0.61 | 1.22 | 0.58 | 1.71 | |
| No | 99.39 | 98.78 | 99.42 | 98.29 | |
| Statins, n(%) | | | | | 0.0121 |
| Yes | 82.65 | 87.93 | 82.35 | 83.76 | |

| | | | | | |
|---|---|---|---|---|---|
| No | 17.35 | 12.07 | 17.65 | 16.24 | |
| Hepatitis B, n(%) | | | | | <0.0001 |
| Yes | 1.25 | 1.80 | 1.64 | 3.55 | |
| No | 98.75 | 98.20 | 98.36 | 96.45 | |
| Hepatitis C, n(%) | | | | | <0.0001 |
| Yes | 1.06 | 0.24 | 1.46 | 6.03 | |
| No | 98.94 | 99.76 | 98.54 | 93.97 | |
| AST（U/L） | 26.04 ± 10.41 | 25.38 ± 16.32 | 24.85 ± 10.15 | 30.09 ± 25.75 | <0.0001 |
| ALT（U/L） | 24.82 ± 15.66 | 24.48 ± 14.54 | 24.93 ± 14.56 | 28.62 ± 26.88 | <0.0001 |
| GGT（U/L） | 26.29 ± 27.69 | 25.53 ± 32.51 | 25.23 ± 21.24 | 46.95 ± 91.90 | <0.0001 |
| ALP (U/L) | 69.11 ± 38.62 | 71.46 ± 40.62 | 72.11 ± 40.67 | 72.64 ± 30.06 | 0.0242 |
| TBIL（μmol/L） | 10.81 ± 6.88 | 10.44 ± 4.99 | 10.53 ± 4.48 | 9.38 ± 4.14 | <0.0001 |
| HDL（mmol/L） | 54.77 ± 18.00 | 52.29 ± 14.52 | 51.35 ± 15.36 | 50.23 ± 15.83 | <0.0001 |
| Total cholesterol（mmol/L） | 186.89 ± 40.65 | 181.75 ± 43.00 | 179.66 ± 40.33 | 187.98 ± 42.82 | <0.0001 |
