# Supplementary material for: Associations between Ethylene Oxide Exposure and Liver Function in the US Adult Population
Source: Toxics. 2024 Jul 30;12(8):551. doi: 10.3390/toxics12080551 (PMC11358929; doi:10.3390/toxics12080551)
Supplement: Supplementary file 1 [file toxics-12-00551-s001.zip › Table S2.pdf]

Table S2 The threshold effect of log2-HbEO on LFTs using a two-stage phased regression model

Threshold effect analysis of Ethylene oxide on ALP using the two-piecewise linear regression model

Gamma-glutamyltransferase	Adjusted β (95% CI) P value
Fitting by the standard linear model	2.25 (1.63, 2.87)
Inflection point	6.73
Log2 Ethylene oxide<6.73（pmol/g Hb）	0.54 (-0.47, 1.55) 0.2918
Log2 Ethylene oxide>6.73（pmol/g Hb）	7.24 (4.83, 9.65) <0.0001
Log likelihood ratio	<0.001

Threshold effect analysis of Ethylene oxide on ALT using the two-piecewise linear regression model

Gamma-glutamyltransferase	Adjusted β (95% CI) *P* value
Fitting by the standard linear model	0.68 (0.23, 1.13)
Inflection point	6.58
Log2 Ethylene oxide<6.58（pmol/g Hb）	-0.41 (-1.16, 0.35) 0.2884
Log2 Ethylene oxide>6.58（pmol/g Hb）	3.43 (1.83, 5.03) <0.0001
Log likelihood ratio	<0.001

Threshold effect analysis of Ethylene oxide on AST using the two-piecewise linear regression model

Gamma-glutamyltransferase	Adjusted β (95% CI) *P* value
Fitting by the standard linear model	-0.21 (-0.72, 0.30)
Inflection point	6.62
Log2 Ethylene oxide<7.97（pmol/g Hb）	-1.82 (-2.67, -0.97) <0.0001
Log2 Ethylene oxide>7.97（pmol/g Hb）	4.02 (2.16, 5.89) <0.0001
Log likelihood ratio	<0.001

Threshold effect analysis of Ethylene oxide on GGT using the two-piecewise linear regression model

Gamma-glutamyltransferase	Adjusted β (95% CI) *P* value
Fitting by the standard linear model	5.48 (4.22, 6.74)
Inflection point	6.08
Log2 Ethylene oxide<6.08（pmol/g Hb）	-2.10 (-4.42, 0.23) 0.0769
Log2 Ethylene oxide>6.08（pmol/g Hb）	17.81 (14.38, 21.23) <0.0001
Log likelihood ratio	<0.001
