# Supplementary material for: Associations between Ethylene Oxide Exposure and Liver Function in the US Adult Population
Source: Toxics. 2024 Jul 30;12(8):551. doi: 10.3390/toxics12080551 (PMC11358929; doi:10.3390/toxics12080551)
Supplement: Supplementary file 1 [file toxics-12-00551-s001.zip › Table S3.pdf]

Supplementary Table S3. The association between HbEO and the liver function test.

LFTs	Model	Continuous EO	Quartile 1<br>*β*(95%CI)	Quartile 2<br>*β*(95%CI)	Quartile 3<br>*β*(95%CI)	Quartile 4<br>*β*(95%CI)	*P* for trend
ALP	Crude	1.00(0.40, 1.60) 0.0012	0.00（Ref.）	2.35 (-0.06, 4.75) 0.0556	3.00 (0.47, 5.53) 0.0201	3.53 (1.06, 6.00) 0.0051	0.0179
	Model 1	0.47(-0.12, 1.06) 0.1169	0.00（Ref.）	1.98 (-0.36, 4.31) 0.0974	2.97 (0.48, 5.46) 0.0194	1.68 (-0.73, 4.10) 0.1722	0.4080
	Model 2	**2.61(1.97, 3.24) <0.0001**	0.00（Ref.）	0.48 (-1.78, 2.74) 0.6760	3.75 (1.30, 6.20) 0.0027	**8.48 (5.75, 11.22) <0.0001**	<0.0001
ALT	Crude	0.72(0.42, 1.01) <0.0001	0.00（Ref.）	-0.34 (-1.51, 0.84) 0.5754	0.11 (-1.12, 1.35) 0.8573	3.80 (2.59, 5.01) <0.0001	<0.0001
	Model 1	0.69(0.40, 0.99) <0.0001	0.00（Ref.）	-0.42 (-1.57, 0.73) 0.4747	-0.73 (-1.96, 0.49) 0.2398	**3.65 (2.46, 4.83) <0.0001**	<0.0001
	Model 2	**0.50 (0.09, 0.90) 0.0158**	0.00（Ref.）	-1.65(-3.07, -0.24) 0.0223	-0.25 (-1.79, 1.29) 0.7517	0.36 (-1.36, 2.07) 0.6846	0.3020
AST	Crude	0.67(0.40, 0.94) <0.0001	0.00（Ref.）	-0.67 (-1.73, 0.40) 0.2187	-1.20 (-2.32, -0.08) 0.0358	4.05 (2.95, 5.14) <0.0001	<0.0001
	Model 1	0.71(0.44, 0.98) <0.0001	0.00（Ref.）	-0.67 (-1.72, 0.39) 0.2136	-1.71 (-2.84, -0.59) 0.0028	4.16 (3.07, 5.25) <0.0001	<0.0001
	Model 2	-0.39 (-0.89, 0.11) 0.1242	0.00（Ref.）	-2.54(-4.29, -0.78) 0.0047	-3.17 (-5.07, -1.26) 0.0011	**-2.60 (-4.73, -0.48) 0.0165**	0.0695
GGT	Crude	4.53(3.72, 5.35) <0.0001	0.00（Ref.）	-0.75 (-3.98, 2.48) 0.6476	-1.06 (-4.46, 2.35) 0.5430	20.66(17.34,23.98) <0.0001	<0.0001
	Model 1	4.62(3.80, 5.44) <0.0001	0.00（Ref.）	-1.09 (-4.31, 2.12) 0.5049	-2.70 (-6.13, 0.72) 0.1222	20.81(17.49,24.14) <0.0001	<0.0001
	Model 2	**5.75 (4.46, 7.05) <0.0001**	0.00（Ref.）	-3.84 (-8.43, 0.76) 0.1018	-1.33 (-6.32, 3.66) 0.6016	**15.06 (9.50, 20.63) <0.0001**	<0.0001
TBIL	Crude	-0.39(-0.48, -0.31) <0.0001	0.00（Ref.）	-0.38(-0.71, -0.04) 0.0302	-0.29 (-0.64, 0.07) 0.1150	-1.43 (-1.78, -1.08) <0.0001	<0.0001
	Model 1	-0.42(-0.50, -0.34) <0.0001	0.00（Ref.）	-0.42(-0.75, -0.09) 0.0125	-0.62 (-0.98, -0.27) 0.0005	-1.55 (-1.89, -1.21) <0.0001	<0.0001
	Model 2	**-0.30 (-0.43, -0.16) <0.0001**	0.00（Ref.）	0.18 (-0.29, 0.65) 0.4593	0.10 (-0.41, 0.61) 0.7009	**-1.19 (-1.76, -0.62) <0.0001**	<0.0001

Model 1: no covariates were adjusted.

Model 2: age, gender, and race were adjusted.

Model 3: age, gender, race, educational level, BMI, family income-to-poverty ratio, moderate activities, diabetes status, smoking, aspirin, acetaminophen, alcohol, statins, hepatitis B, hepatitis C, HDL level, Total cholesterol were adjusted
